# Response to the Letter to the Editor by Rob Armstrong

**DOI:** 10.1186/s13071-017-2142-4

**Published:** 2017-04-26

**Authors:** Robert H. Six, David R. Young, Melanie R. Myers, Sean P. Mahabir

**Affiliations:** 1 Zoetis, Veterinary Medicine Research and Development, 333 Portage St, Kalamazoo, MI 49007 USA; 2 YVRS, 7243 East Ave, Turlock, CA 95380 USA

## Abstract

In a recent Letter to the Editor, Armstrong raises concern that the design of the study reported by Six et al. was not consistent with the product label for treatment of *Amblyomma americanum*, since fluralaner was not re-administered 56 days after the initial treatment. The Authors disagree with this assessment and confirm that the design was appropriate, and therefore the results and conclusions for the entire study period are valid.

## Letter to the Editor

Six et al. reported the results of a study demonstrating that monthly administration of sarolaner consistently killed significantly more *A. americanum* ticks within 24 h than a single dose of fluralaner from 6 weeks after initial treatment [1]. In his Letter to the Editor, Armstrong raised concern that the efficacy of fluralaner was evaluated at times outside the label administration recommendations [2].

We appreciate the author’s review and comments on the manuscript; however, there are several points that need to be addressed in order to appropriately evaluate the conclusions they have drawn:

(i.) The author states that the approved Bravecto® label *requires* that fluralaner be re-administered at 56 days for the treatment *A. americanum* when in fact the label [3, 4] states that fluralaner *may* be re-administered every 8 weeks in case of potential exposure to *A. americanum* (although the general dosing directions state fluralaner *should* be administered every 12 weeks). This language is very confusing and may be interpreted by a pet owner or veterinarian to mean that fluralaner may be effective against *A. americanum* for up to 12 weeks. This supports the study design which evaluated efficacy for 12 weeks and therefore we believe the results and conclusions for the entire study period are valid.
(ii.) The author states that the manuscript reports in the Discussion section that the Bravecto® label directions were not followed when in fact the Discussion section simply references the efficacy of fluralaner against *A. americanum* as reported in the Bravecto® Freedom of Information Summary [3]; the design of the study (a single dose of fluralaner compared to 3 monthly doses of Simparica® over a 90 day period) is clearly stated in the Methods section of the Abstract.
(iii.) The author accurately summarizes the manuscript which reported that at the 8 and 12 h counts on Day 42 the efficacy of both products was lower than expected for unknown reason; however, at the 24 h count on Day 42 and for the duration of the study through Day 90, sarolaner provided significantly lower live tick counts compared to fluralaner (*P* ≤ 0.0006) [1].

The design, conduct and reporting of this study are therefore accurate and appropriate. Under the conditions of this study, monthly administration of Simparica® provided consistently high (> 70%) efficacy against *A. americanum* within 24 h of re-infestations over the 3-month (3 dose) period and from Day 42 onwards this efficacy was significantly better than that provided by fluralaner.

Veterinarians and pet owners cannot predict at the time of treatment the genera and species of tick to which a dog will potentially be exposed. This consideration is of even greater importance when a long-acting product (where the tick genera and species must be predicted months in advance) is used. With Bravecto® this is further complicated by the fact that the duration of efficacy and therefore the ideal treatment interval varies depending upon the tick species. The design of the study is consistent with the fluralaner re-treatment interval recommended on the product label (anywhere between 8 and 12 weeks), and demonstrates the efficacy observed under controlled conditions from a re-treatment interval that will possibly be used on dogs exposed to *A. americanum*.

This study therefore clearly demonstrates that the rapid and consistent kill of *A. americanum* provided by monthly dosing with Simparica® will reduce the direct deleterious effects of tick feeding and should reduce the risk of tick-borne disease transmission better, and more reliably than a single dose of fluralaner over a 90-day period.
